# The Engagement of the Pelvic Floor Muscles to the Urethra, Does Variation in Point of Action Exist?

**DOI:** 10.3389/fped.2019.00522

**Published:** 2020-01-08

**Authors:** Frank-Jan van Geen, Henriëtte M. Y. de Jong, Tom P. V. M. de Jong, Keetje L. de Mooij

**Affiliations:** ^1^Department of Social Medical Affairs, UWV, The Hague, Netherlands; ^2^Department of Pediatric Urology, University Children's Hospitals UMC Utrecht and Amsterdam UMC, Amsterdam, Netherlands

**Keywords:** pelvic floor anatomy, puborectalis muscle, urethra, dynamic ultrasound, anatomical variation, female

## Abstract

**Purpose:** Lower urinary tract dysfunction (LUTD) occurs frequently in girls and may display a spinning top urethra (STU) on voiding cysto-urethrogram (VCUG) in case of dysfunctional voiding. A STU presents as a narrowing of the urethra caused by a lack of relaxation of the pelvic floor musculature during micturition and may vary in length between the proximal and the distal urethra. Although a STU has been recognized since 1960 as a pathological entity on VCUG, no reports exist on the different levels of engagement of the pelvic floor muscles to the urethra as expressed by the varying length of the phenomenon. The aim of our study is to demonstrate the wide anatomical variation in the level of engagement of the pelvic floor musculature to the urethra.

**Materials and Methods:** Dynamic ultrasound videos of pelvic floor musculature of 40 girls with LUTD were reassessed by three observers, looking for the level of engagement of the puborectalis muscle (PRM) to the urethra during coughing, Valsalva and hold-up maneuver. Three levels were defined, for the level of engagement of the pelvic floor to the urethra, proximal, mid, and distal. Intra- and inter-rater variability was analyzed using Cohen's kappa statistics.

**Results:** A wide range of points of action was found on the assessed ultrasound videos. Intra- and inter-rater agreement showed different levels of conformity, varying over a wide spectrum (intra-rater kappa 0.145–0.546; inter-rater kappa −0.1030.724). Throughout the assessed videos, all not-corresponding intra-rater observations differed maximal one category. Of the not-corresponding inter-rater observations, 90% differed maximal one category.

**Conclusion:** An anatomical variation in levels of engagement of the PRM to the urethra does exist. The clinical value of this finding, whether the point of engagement influences symptomatology or treatment success of LUTD, is currently being studied.

## Introduction

From pediatric and radiologic literature on VCUG we have learned that a spinning top urethra (STU) may be seen in girls with LUTD (1–3), diagnosed as dysfunctional voiding, who are not able to properly relax their pelvic floor during micturition (3–5). This widely used term reflects a marked dilatation of the proximal part of the urethra (3–5), up to the level of engagement of the puborectalis muscle (PRM), a finding that has been recognized as a pathological entity during VCUG for nearly five decades now (6). Firstly, the narrowing of the urethra was considered to be an anatomical obstruction with abundant literature to cure this by dilatation or urethrotomy (7). The dilatation and internal urethrotomy that had been advised was later named as a barbaric procedure (8). During the eighties of the last century literature describing non-neurogenic functional problems of the lower urinary tract came up culminating in the first standardization report in 1998 (9). It has learned us that the constriction of the urethra in the VCUG with STU does not represent a membrane but a functional constriction by the pelvic floor musculature, with the PRM in direct contact with the rectum, vagina, and urethra (1–4, 10).

**Figure 1:**
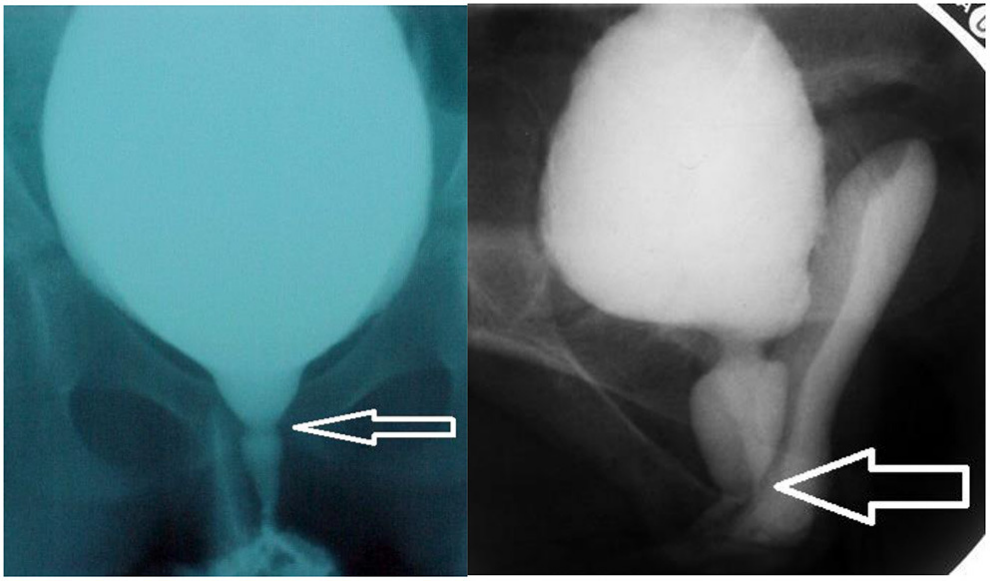
**(A)** (left) proximal spinning top urethra and **(B)** distal STU. Two examples of VCUG pictures with spinning top urethra (STU), one with a short STU with proximal constriction of the pelvic floor musculature, one with a distal constriction and a long STU.

From experience, we know that the length of the STU on VCUG may vary between the proximal and distal urethra, an observation that is suggestive for the existence of different levels of point of action of the pelvic floor musculature to the urethra (Figure 1). Literature on this phenomenon is very sparse (7, 10, 11).

**Figure 2:**
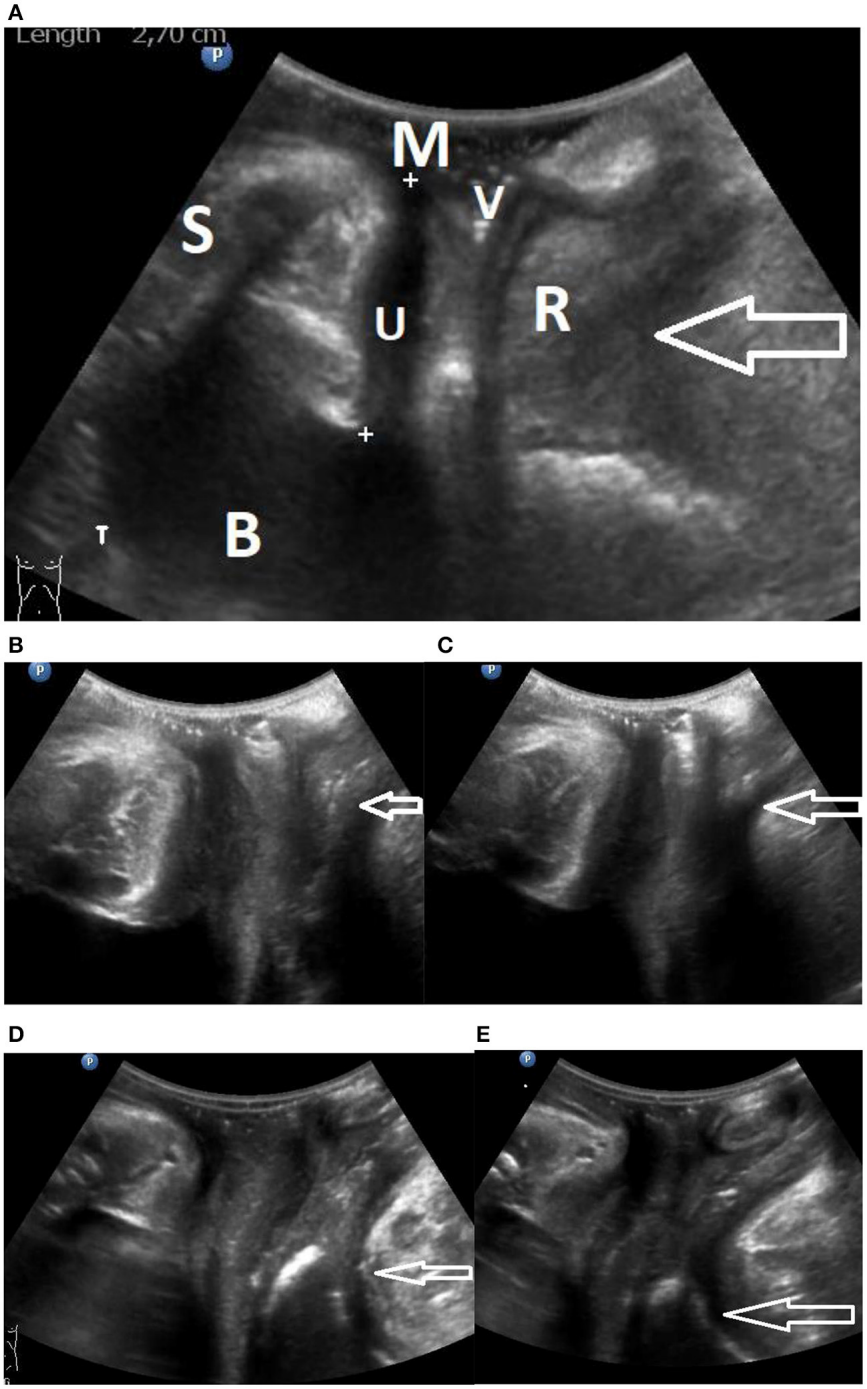
**(A)** Gives an overview of a perineal ultrasound image. B, bladder; U, urethra; V, vagina; R, rectum; M, meatus; S, symphysis. The point of the arrow is in the puborectalis muscle. **(B,C)** Snapshots from a video relaxed and contracted. Distal engagement of the PRM, **(B)** relaxed and **(C)** contracted state, arrows point at PRM engagement. Videos are uploaded as proximal video and distal video. **(D,E)** Snapshots from a video relaxed and contracted. Proximal engagement of the PRM, **(D)** relaxed state, **(E)** contracted state. Arrows point at PRM engagement.

Over years, we have assessed the function of the pelvic floor musculature (PFM) and the urethra by perineal dynamic ultrasound (US) of the pelvic floor during coughing, straining (Valsalva) and when performing a hold-up maneuver (try to hold back your micturition by contracting your pelvic floor muscles). During contraction of the PFM and sphincter muscles a lengthening and anterior displacement of the urethra and compression of the vagina and rectum can be observed (10, 12, 13). A comprehensive overview of the anatomy and dynamic function of the pelvic floor musculature is given by Chamie et al. (10). Figure 2 is a schematic drawing of the PRM in relationship to the rectum, vagina, and urethra.

**Figure 3:**
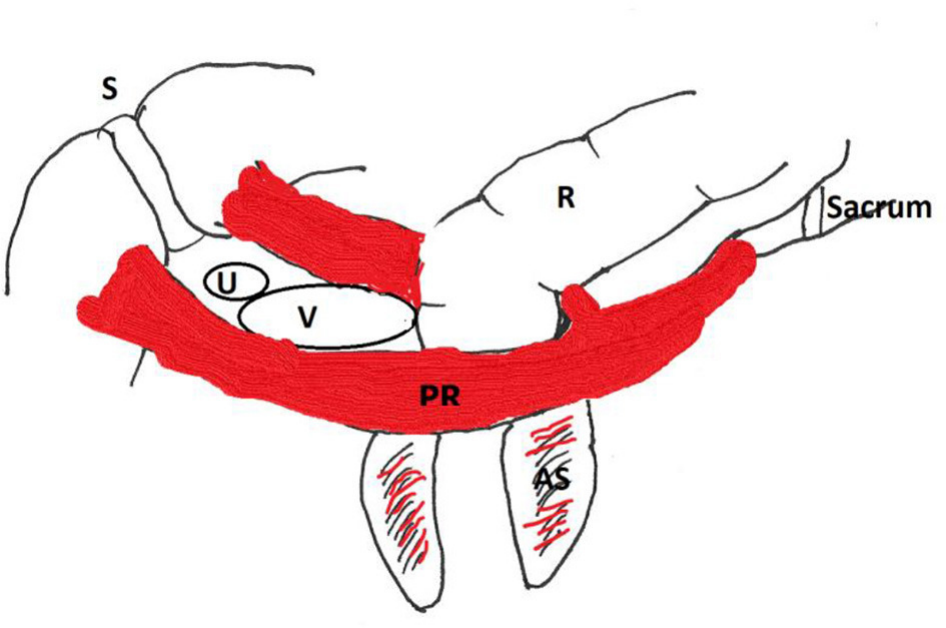
Schematic drawing. S, Symphysis; R, rectum; PR, puborectalis muscle; V, vagina; U, urethra. AS, anal sphincter.

The aim of our study is to demonstrate the existence of a wide range of points of action considering the level of engagement of the PRM to the urethra. Figure 2A gives an overview of the aspect of the pictures that are observed; Figures 2B–E show the difference between a distal and a proximal engagement of the PRM in relaxed and contracted state. Two corresponding videos showing contraction of the PFM in a case of proximal engagement and a case of distal engagement are uploaded as proximal and distal (Supplementary Videos 1, 2). Figure 3 is a schematic drawing of the anatomy.

## Materials and Methods

In our 3rd line referral center for children with LUTD refractory to treatment in the second line, as part of our standard protocol, a perineal US is performed to assess the pelvic floor function in children. Urethra, bladder neck, and pelvic floor muscle function are routinely observed while resting, during coughing, when performing Valsalva and during a hold-up maneuver (12, 13). When making a dynamic video the US stores 140 pictures in 3 s. The pictures and videos are stored in the electronic patient data system and can be retrieved for review at any time.

We retrospectively re-assessed dynamic ultrasound videos of 40 school age girls with refractory LUTD that were referred to our pediatric incontinence university clinic between the years 2010–2016. Age at US assessment was between 9 and 15 years. They all had earlier unsuccessful urotherapy and pharmacotherapy in outpatient programs in general hospitals, for LUTD diagnosed as dysfunctional voiding and/or overactive bladder. The vast majority (33) was at prepubertal age and menstrual status has not been recorded routinely. Inclusion was determined by the availability of a complete set of US pictures and at least 3 videos of sufficient quality for reassessment.

The level of engagement of the PRM upon the urethra was assessed on the existing US videos. Contraction of the PFM on the videos can be observed during hold-up maneuver, Valsalva, and coughing. Three different observers (a pediatric urologist with abundant US experience and two non-specialists MD) classified independently the level of engagement of the PRM to the urethra. US videos were reviewed in randomized order and assessed during two reading sessions with unlimited reading time available, a minimum of 14 days existing in-between readings.

US was done by placing a 7 Mhz convex probe, covered by a protective sleeve, directly on the urethral meatus with the patient in supine position. A Philips HD11XE^®^ US system has been used.

Prior to the independent video assessment, the pediatric urologist trained the other observers in reviewing dynamic US videos. Points of action were predefined in three thirds; proximal-, mid-, and distal urethra, indicating the level of engagement of the PRM to the urethra. During contraction of the pelvic floor and sphincteric muscles an elongation and compression of the urethra is to be expected. The level of compression of the urethra is the point of engagement of the PRM and can be seen varying from the bladder neck to the distal urethra near the meatus.

## Outcome Measures

Primary end-point was the existence of variability in the level of engagement of the PRM to the urethra. Intra- and inter-rater variability were analyzed using Cohen's kappa statistics. Kappa values were defined as follows: slight: 0.00–0.20; fair: 0.21–0.40; moderate: 0.41–0.60; substantial: 0.61–0.80; and almost perfect alignment: 0.81–1.00 (14). Statistical analyses have been performed using IBM Statistical Package for Social Sciences software (version 21, SPSS).

The Institutional Ethical Committee consented with the followed procedure (reference number WAG/mb/19/020335).

## Results

Results on the dynamic ultrasound video assessment of the 40 included patients are presented in Table 1. All three predefined categories of points of action were independently visualized by all observers, although in different frequencies. Results show the distribution of variation in level of engagement on the urethra, throughout the different readings by different observers.

**Table 1:** Variation is points of action classification.

***N = 40***	**Proximal engagement**	**Mid engagement**	**Distal engagement**
Observer 1; reading 1 (Pct. of total)	*N =* 6 (15%)	*N =* 28 (70%)	*N =* 6 (15%)
Observer 1; reading 2 (Pct. of total)	*N =* 23 (57.5%)	*N =* 11 (27.5%)	*N =* 6 (15%)
Observer 2; reading 1 (Pct. of total)	*N =* 9 (22.5%)	*N =* 20 (50%)	*N =* 11 (27.5%)
Observer 2; reading 2 (Pct. of total)	*N =* 8 (20%)	*N =* 24 (60%)	*N =* 8 (20%)
Observer 3; reading 1 (Pct. of total)	*N =* 18 (45%)	*N =* 16 (40%)	*N =* 6 (15%)
Observer 3; reading 2 (Pct. of total)	*N =* 14 (35%)	*N =* 20 (50%)	*N =* 6 (15%)

Cohen's kappa was utilized to determine intra- and inter-rater agreement. Table 2 shows the outcome for all individual observers when the different reading sessions are compared to each other. Intra-rater conformity between the two different sessions varies from 40% (*kappa* 0.145) to 72.5% (*kappa* 0.546). Inter-rater variability through comparison of the different reading sessions performed by the observers is presented in Table 3. Results are displayed as agreement percentage with coordinating Cohen's kappa. Herein a wide range in conformity is found, with values varying from 27.5% (*kappa –*0.103) to 85% (*kappa* 0.724).

**Table 2:** Intra-rater agreement.

***N = 40***	**Observer 1**	**Observer 2**	**Observer 3**
No. of agreement (Pct. of total)	*N =* 16 (40%)	*N =* 29 (72.5%)	*N =* 22 (55%)
Cohen's kappa	0.145	0.546	0.274

**Table 3:** Inter-rater agreement.

***N=40***		**Observer 3**		**Observer 2**	
		**Reading 1**	**Reading 2**	**Reading 1**	**Reading 2**
Observer 1	Reading 1	32.5%	62.5%	77.5%	85%
	(Cohen's kappa)	(−0.61)	(0.358)	(0.612)	(0.205)
	Reading 2	65%	60%	45%	52.5%
	(Cohen's kappa)	(0.425)	(0.452)	(0.205)	(0.315)
Observer 2	Reading 1	27.5%	55%	X	X
	(Cohen's kappa)	(−0.103)	(0.286)		
	Reading 2	37.5%	57.5%	X	X
	(Cohen's kappa)	(0.033)	(0.302)		

To determine the scope of the non-conforming observations, we subsequently assessed our data. We hereby determined that 90% (*n* = 36) of the non-conforming observations differed maximal one category when classified points of action were compared. This implies that an observation could change from proximal into mid or mid into distal, not from proximal into distal.

## Discussion

Our results show that the PRM have different points of engagement to the urethra. All observers reported independently on the existence of different points of action. We found that the level of agreement differed between the various observers and reading sessions. The diverse levels of agreement suggests that assessing the exact level of engagement is challenging, but underscores the finding that different levels of engagement do exist. In all intra-rater cases and in the vast majority (90%) of inter-rater cases scoring differences were to the utmost 1 category, that is proximal instead of mid or mid instead of distal.

To our best knowledge, this is, after a histological suggestion done in 1986, the first report on the existence of anatomical variation with different points of actions of the PRM on the urethra (11). Therefore, no reference standard was available for assessing the levels of engagement of the PRM onto the urethra. We acknowledge this as one of our shortcomings as it may have resulted in an intra- and inter-rater variability.

The clinical relevance to do dynamic ultrasound of the lower urinary tract lies in the fact that the vast majority of our refractory patients did have earlier pelvic floor physical therapy. By looking at the conscious command of the PFM we can detect those subjects with apraxia of the PFM in need for specific physical therapy with anal balloon biofeedback. Of course, one can discuss whether this study is focussed specifically on the PRM or that we should call it a study on the pelvic floor musculature, since the PRM is part of the levator ani and pelvic floor musculature as a whole. It is not exactly possible to discriminate, by US, between the puborectalis and the pubococcygeus muscle. Chamie et al. (10), published an elegant study with, other than US, also MRI pictures and videos that may be used as a control study and justifies the use of the term PRM in our report.

Although we report on the existence of different points of action, our result do not provide an answer to the clinical relevance of this observation in children with LUTD. We tried to demonstrate the wide variability, but did not assess the difference in symptomatology or treatment success. The study has been initiated by the fact that we had an impression that those girls with a distal engagement of the PRM performed worse in urotherapy. This impression could not be confirmed in this relatively small group of patients.

Limitations of the study are the fact that it has been a retrospective study and that 2 of 3 reviewers of the US reassessment were relatively unexperienced MD's.

## Conclusion

The results of this study have demonstrated that different points of action of the PRM can be observed on dynamic ultrasound videos. Therefore, we conclude that anatomical variation with different levels of engagement on the urethra does exist. The clinical value of this finding, whether the point of engagement influences symptomatology or treatment success of LUTD, is subject of an on-going study. The relatively poor inter-and intra-observer levels of agreement illustrate that correct interpretation of the pictures is difficult, but the different observations vary maximal one level, thus not influencing the final conclusion.

## Data Availability Statement

The datasets generated for this study are available on request to the corresponding author.

## Ethics Statement

The studies involving human participants were reviewed and approved by Medical Ethical committee University Medical Center Utrecht. Written informed consent from the participants' legal guardian/next of kin was not required to participate in this study in accordance with the national legislation and the institutional requirements.

## Author Contributions

TJ: design of the study, instruction of co-authors to assess ultrasound videos, and editing of the manuscript. F-JG: assessment of ultrasound video's, statistic evaluation, and preparation and writing the manuscript. HJ: assessment of ultrasound videos and preparation and writing the manuscript. KM: collection of patients and patient material, codesigner of the study, and literature search.

### Conflict of Interest

The authors declare that the research was conducted in the absence of any commercial or financial relationships that could be construed as a potential conflict of interest.

## Supplementary Material

Supplementary Video 1 Shows a contraction of a distal engagement of the PRM.

Supplementary Video 2 Shows a contraction of a proximal engaged PRM.

## Abbreviations

PRM: puborectalis muscle
PFM: pelvic floor muscles
STU: spinning top urethra
VCUG: voiding cysto-urethrogram
LUTD: lower urinary tract dysfunction
US: ultrasound.
